# Limonene Selectively Modulates Visual Attention Through P300 Suppression: A Comparative Event‐Related Potential Study With Lemon Essential Oil

**DOI:** 10.1002/brb3.71012

**Published:** 2025-10-29

**Authors:** Kaori Tamura, Taiki Nishimura, Yuko Ohno, Shin'ichi Yoshimura, Mitsuo Tonoike

**Affiliations:** ^1^ Department of Basic Sciences, Faculty of Engineering Kyushu Institute of Technology Kitakyushu Japan; ^2^ Graduate School of Engineering, Fukuoka Institute of Technology Fukuoka Japan; ^3^ Division of Health Sciences, Graduate School of Medicine The University of Osaka Suita Japan; ^4^ Department of Systems Innovation, Graduate School of Engineering Science The University of Osaka, Suita Japan

**Keywords:** event‐related potentials, limonene, P300, visual selective attention

## Abstract

**Introduction:**

Odor stimuli can influence cognitive processes, including selective attention. However, whether these effects are driven by the chemical properties of odor compounds or by their semantic or emotional associations remains unclear. This study aimed to clarify the mechanism by which olfactory inputs influence visual attention by isolating the effects of specific odor compounds from those of affective or semantic factors.

**Methods:**

To investigate this question, we assessed the effects of limonene, a key compound found in citrus odors, and lemon essential oil on visual attention, as indexed by the P300 component of event‐related potentials, which reflect the allocation of selective attention. Participants completed a visual oddball task under three odor conditions: no odor, limonene, and lemon essential oil.

**Results:**

Limonene presentation significantly reduced P300 peak amplitudes compared with the no‐odor condition, whereas lemon essential oil showed not significantly reduce. Subjective ratings of pleasantness, congruency, and arousal did not differ significantly between the two odors.

**Conclusions:**

Our results suggest that limonene modulates selective attention primarily through its chemical properties rather than through mood or semantic influences. This study provides new evidence for chemically specific olfactory–visual interactions and underscores the importance of distinguishing chemical effects from psychological mechanisms in cross‐modal cognitive modulation.

## Graphical abstract

Limonene, a citrus compound, selectively reduced P300 amplitude during a visual oddball task, suggesting a modulation of attention. The effect was not observed with lemon essential oil, indicating that cognitive modulation may stem from limonene's chemical properties rather than semantic or emotional associations.

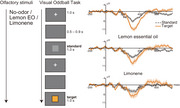

## 1 Introduction

Olfactory–visual interactions have become a central topic in multisensory integration and cognitive enhancement research. The associations between olfactory and visual modalities have been discussed in the context of cross‐modal correspondences, such as the link between lemon odor and yellow color (Demattè et al., 2006; Gilbert et al. 1996; Guerdoux et al. 2014; Russell et al. 2015; Seo et al. 2010; Stevenson et al. 2012; Spence 2020; Walla 2008;). Olfactory determination can be influenced by visual information at both behavioral (Dematte et al. 2009) and neuronal levels (Gottfried and Dolan 2003), particularly when visual inputs are congruent with odors. For instance, Jadauji et al. (2012) reported that transcranial magnetic stimulation applied to the visual cortex enhances olfactory perception, indicating a behavioral and neurophysiological link between visual processing and olfaction.

Beyond these correspondences between the visual and olfactory senses, olfactory input can enhance visual cognition. Conventional studies using event‐related potentials (ERPs) have shown that olfactory stimulation can facilitate visual ERPs when the odor is congruent with the visual information (Bensafi et al. 2013; Grigor et al. 1999; Robinson et al. 2015). Olfactory inputs can support visual cognitive processes (Hörberg et al. 2020; Rekow et al. 2022). Olfactory modulation of visual processing has also been observed at low‐level visual perception, based on visual cortex activity during visual tasks, even when the visual information is not semantically related to odors (Tsushima et al. 2021).

Our previous findings revealed that citrus odor input can modulate visual cognitive processes, as reflected in changes in memory performance and ERP components (Tamura et al. 2018). In that study, we used decanal, a compound found in grapefruit peel, as the odor stimulus. The presentation of decanal inhibited the working memory for the color orange, while other colors were unaffected. During orange retrieval under decanal presentation, the P300 amplitude, an ERP component associated with selective attention and memory updating (Polich 2007), was also reduced. The relationship between P300 and attentional resource allocation has been well investigated, indicating that decanal may interfere with the assignment of selective attention to visual inputs, leading to reduced working memory performance. Notably, a P300 reduction was also observed during the retrieval of pink stimuli; however, memory performance for pink stimuli was not significantly affected by decanal presentation. Furthermore, in this study, mood effects induced by the odor were not sufficiently assessed, nor were the influences of odor‐related mood or visual–odor correspondences on attentional processes or P300 elicitation examined.

Citrus odors have often been associated with enhanced attention and increased arousal levels, and their effects on mood and feelings have also been discussed, although the findings remain controversial. For example, de Wijk and Zijlstra reported that citrus odors reduce negative emotions and increase metabolic equivalents (de Wijk and Zijlstra 2012). Hoenen et al. (2016) observed that limonene, a prominent component of citrus peels, elevated pleasant feelings; however, this effect was attributed to the positive mood associated with odor pleasantness rather than to the odor itself. Conversely, other studies have reported no effects of citrus odors on mood or anxiety levels (Pimenta et al. 2012; Haehner et al. 2017).

Regarding cognitive performance, citrus odor presentation has been shown to improve cognitive task scores and electrodermal activity during the task, although these effects were not accompanied by direct changes in autonomic nervous system activity (Shikha et al.,^2025^). Similarly, Tong et al. (2025) reported that orange odor improved cognitive task performance and attentional level; however, they suggested that these improvements were mediated by a positive mood rather than the odor itself. Kiecolt‐Glaser et al. (2008) reported that the lemon oil odor improved positive mood but did not significantly elevate physiological responses, including increased salivary cortisol production, higher heart rate, or elevated blood pressure. Together, these studies suggest that while citrus odors or limonene can influence cognitive function and attention, it remains unclear whether these effects result directly from the odor or from its semantic or emotional states.

In this study, we investigated whether the modulatory effects of citrus odors on visual attention are attributable to the specific chemical properties of the odorants or arise from their semantic or emotional associations. Specifically, we focused on the effects of olfactory stimulation on P300 elicitation within a visual oddball paradigm. The P300 component of ERPs is characterized by a positive peak occurring approximately 300 ms post‐stimulus and reflects the allocation of attentional resources. The oddball paradigm has been widely employed in P300 research and is known to elicit P300 responses to infrequent target stimuli (Polich 2007). We employed a classical oddball task comprising standard and target visual stimuli, presented under three odor conditions: no odor, limonene, and lemon essential oil. Limonene is a single chemical compound found in lemon peel, while lemon essential oil contains limonene and other compounds, including monoterpene hydrocarbons (such as α‐pinene, β‐pinene, γ‐terpinene, and myrcene), oxygenated monoterpenes, sesquiterpene hydrocarbons, and fatty alcohol esters (Cannon et al. 2015; González‐Mas et al. 2019; Di Rauso Simeone et al. 2020). By comparing pure limonene with lemon essential oil, we aimed to dissociate chemically specific effects from broader psychological influences. Citrus odors have been associated with enhanced attention and arousal previously; however, such effects may be mediated by subjective impressions such as odor pleasantness or odor–color congruency. To address this, we collected subjective ratings of pleasantness, congruency, and arousal for each odor condition. If P300 amplitudes were more strongly suppressed under limonene conditions than under lemon essential oil conditions, it would suggest that the chemical properties of limonene modulate attentional processes. Conversely, if both odors elicited comparable effects on P300 amplitudes, this would imply that the observed cognitive modulation is driven by mood or semantic associations. The aim of this study was to clarify the mechanism by which olfactory inputs influence visual attention by isolating the effects of specific odor compounds from those of affective or semantic factors.

## 2 Materials and Methods

### 2.1 Participants

The Ethics Committee of the Fukuoka Institute of Technology approved this experiment. All participants provided written informed consent in accordance with the Declaration of Helsinki.

We recruited 19 university students (six women, 13 men; mean age ± SD = 23 ± 1.6 years). None of the participants had color vision deficiencies, as confirmed by the Ishihara test. Additionally, no participants exhibited olfactory or neurological impairments, as assessed using a T&T olfactometer (Daiichi Yakuhin Sangyo Co., Ltd., Tokyo, Japan). Because odor detection and discrimination can vary with cultural and linguistic background (Levitan et al. 2014; Majid and Burenhult 2014; Majid et al. 2018), the enrolled individuals were native Japanese speakers with a shared Japanese cultural background.

### 2.2 Odor Stimuli

We used (R)‐(+)‐limonene (Fujifilm Wako Pure Chemical Corporation, Osaka, Japan) and lemon essential oil (RIMO‐Trogen Inc., Fukuoka, Japan) as odor stimuli. During the oddball task, participants wore a nonwoven mask with a sticker containing the applied odor solution. To achieve comparable perceived odor intensities, 25 µL of limonene or 5 µL of lemon essential oil was applied to the sticker. In the no‐odor condition, participants wore a mask with a sticker that contained no odorant. The amounts of odors were determined by two experimenters using a six‐point odor intensity scale (0 = no odor, 5 = extremely strong smell). The experimenters decided that the amounts of odor for each intensity rating were equal between limonene and lemon essential oil.

### 2.3 Task Design

Participants completed a visual oddball task in a dark room under three conditions: no odor, limonene, and lemon essential oil. Each condition comprised two 50‐trial blocks, resulting in six blocks per participant. Two possible sequences for odor presentation were pseudo‐randomly assigned to participants. In one sequence, participants completed the limonene blocks first, followed by the no‐odor blocks, and then the lemon essential oil blocks. In the second sequence, participants completed the lemon essential oil blocks first, followed by the no‐odor blocks and then the limonene blocks. The no‐odor condition was consistently positioned in the middle to serve as a washout between the two odor conditions.

Each block consisted of 50 trials, with the target stimulus (an orange square) appearing in 20% of the trials and the standard stimulus (a grey square) in the remaining 80%. Before starting the task, participants completed a practice session. For each block, they wore a nonwoven mask embedded with an aroma sticker and rated odor intensity prior to stimulus presentation.

During each trial, a fixation cross was presented on a gray background for 1 s, followed by a blank screen lasting 0.5–0.9 s (randomized). Next, either a standard or target stimulus was presented for 1 s. Participants were instructed to click on a mouse only when the target (an orange square) appeared and to count the total number of target occurrences. These instructions were intended to direct selective attention toward the target stimulus.

**FIGURE 1:**
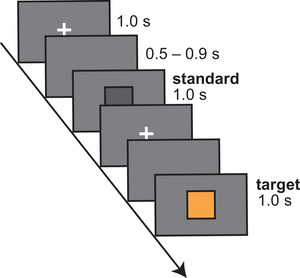
**Visual oddball paradigm**. A schematic illustration of the visual oddball task is shown. The target stimulus (orange) appeared in 20% of 100 trials. Participants were required to count the number of target stimulus appearances and click the mouse only when the target stimulus was displayed. This sequence was repeated three times for the odor conditions: no‐odor, limonene, and lemon essential oil.

Participants were instructed to blink only during the fixation cross period (Figure 1). After each block, they re‐evaluated the odor intensity and rated the pleasantness, preference, arousal, and color–odor congruency between the orange stimulus and presented odor. Odor intensity was assessed using a six‐point scale ranging from 0–5, where 0 = no odor, 1 = barely detectable (detection threshold), 2 = weak but recognizable (recognition threshold), 3 = easily noticeable, 4 = strong, and 5 = extremely strong. Odor pleasantness, preference, and arousal were assessed using separate 11‐point scales, ranging from −5 (not at all pleasant/preferred/arousal) to +5 (extremely pleasant/preferred/arousal). A rating of 0 indicated a neural response. Color–odor congruency for each participant was determined by asking the participant this question: “Does the color as the target stimulus match the color you associated with the odor presented just before? To what extent?” with the response rated on an 11‐point scale from –5 (completely different) to +5 (perfectly matching). All questions and scales were written in Japanese. To maintain a consistent odor strength across blocks, a new mask with a freshly applied odor solution was used after each rest period. All visual stimuli were presented on a 24‐inch liquid‐crystal display monitor in a darkened room, using Psychtoolbox‐3 (Brainard 1997; Kleiner et al. 2007) programmed in MATLAB (MathWorks, Inc., Natick, MA, USA). The monitor was calibrated according to standard procedures (Brainard et al. 2002) using a ColorCAL MKII Colorimeter (Cambridge Research Systems Ltd., Rochester, UK). The target and standard colors were defined in the CIEL*b*a* color space (target: L* = 71.1, a* = 9.1, b* = 64.1; standard: L* = 71.1, a* = 0.0, b* = 0.0) of the calibrated display.

### 2.4 EEG Recording

Electroencephalogram (EEG) data were recorded from 19 channels following the international 10–20 System, using the Polymate Pro MP6100 and wet active electrodes (Miyuki Giken Co., Ltd., Tokyo, Japan). The body‐earth electrode was placed at the frontopolar midline, and the reference electrode was positioned at A1 (left earlobe), as defined by the above system. An additional electrode was placed at A2 (right earlobe) to serve as an offline reference. The data were amplified and digitized at a sampling rate of 500 Hz. The impedance level was maintained below 10 kΩ.

### 2.5 EEG Preprocess and Analysis

EEG data were referenced to A1 during acquisition and re‐referenced offline to the average of A1 and A2. Signals were bandpass filtered between 0.1 and 50 Hz using a finite impulse response filter. Epochs were rejected if amplitudes exceeded ±80 µV within the −200 to 800 ms window. Segmentation was conducted using the visual stimulus onset as the time‐zero marker, and epochs were averaged for each participant. Baseline correction was applied using the −200 to 0 ms window. All preprocessing and averaging were performed using EEGLAB (Delorme and Makeig 2004) and ERPLAB (Lopez‐Calderon and Luck 2014).

### 2.6 Statistics

The peak amplitude of P300 within 250–400 ms was assessed by two‐way ANOVA for within‐subject analysis (odor condition × stimulus type) at the Pz electrode. For post hoc testing, we performed Tukey's honestly significant difference tests for the interested comparisons: no‐odor versus limonene conditions and no‐odor versus lemon essential oil conditions. These tests were conducted using JMP Pro 18 (SAS Insittute Inc., Cary, NC, USA). Effect sizes were calculated using Cohen's *d* according to Cohen's criteria (Cohen 1988).

For the multiple comparisons of P300 peaks, we also conducted Bayesian *t*‐tests using the JZS prior (Cauchy scale *r* = 0.707, medium). According to the mean differences (Figure 4), the primary hypotheses were directional paired comparisons on within‐subject differences (limonene condition—no‐odor condition < 0, lemon essential oil condition—no‐odor condition < 0). The analysis was performed in R and R studio with the BayesFactor package and ttestBF function. Bayes factors were interpreted following conventional heuristics (3–10: moderate; 10–30: strong; ≥30: very strong). BF_10_ was reported for evidence in favor of the alternative over the null and BF_01_ for the reverse.

The subjective ratings of odor intensity between limonene and lemon essential oil were assessed by paired *t*‐test in Table 1 without multiple comparison. The intensity ratings were also assessed by two‐way ANOVA for within‐subject analysis (odor condition × assessment timing: before/after block 1 and 2). Odor pleasantness, preference, arousal, and color‐matching ratings were assessed by paired *t*‐test to compare limonene and lemon essential oil conditions.

**TABLE 1:** Subjective odor intensity of odor conditions.

Odor	Block	Before (*M* ± SD)	After (*M* ± SD)	Mean Diff	*t*	*p*	Cohen's *d*
Limonene	Block 1	3.68 ± 0.67	2.95 ± 0.52	−0.74	−4.38	<0.001	1.05
Lemon	Block 1	3.47 ± 0.61	2.74 ± 0.87	−0.74	−4.38	<0.001	1.05
Limonene	Block 2	3.05 ± 0.52	2.84 ± 0.60	−0.21	−1.46	0.163	0.34
Lemon	Block 2	3.21 ± 0.42	2.63 ± 0.59	−0.58	−3.64	0.002	0.84

*Note*: Mean subjective odor intensity ratings before and after each experimental block under different odor conditions. Ratings were assessed on a six‐point scale from 0 (no odor) to 5 (extremely strong odor). Mean values of before and after ratings were compared between limonene and lemon essential oil conditions by paired *t*‐test in each column.

## 3 Results

### 3.1 ERP

**FIGURE 2:**
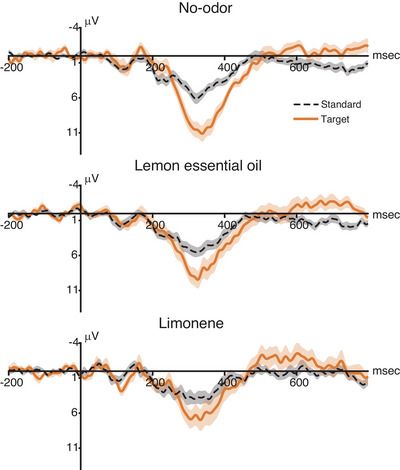
**Event‐related potentials waveform at the Pz (located at the midline parietal region of the scalp) electrode**. Grand‐average ERP waveforms at the Pz electrode across participants. Bold lines represent mean amplitudes, and the shaded areas indicate the standard error of the mean. Waveforms in response to target stimuli are shown with solid lines, while those for standard stimuli are depicted with a dotted line. Stimulus onset is aligned at time zero.

**FIGURE 3:**
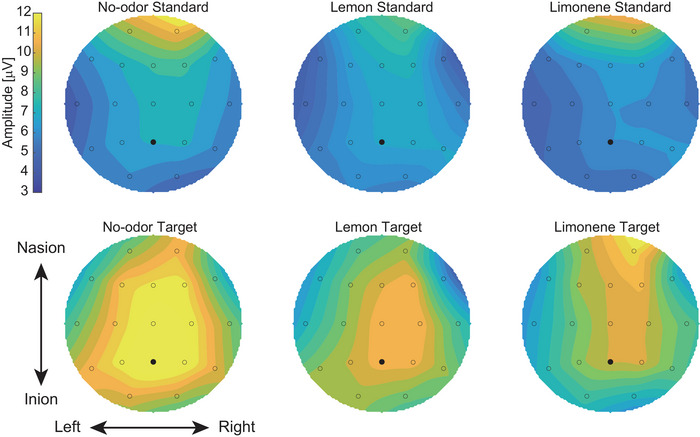
**Event‐related potential topography of mean P300 peak**. Scalp topographies show the mean P300 peak amplitude across the 250–400 ms window for each odor condition. Responses to target and standard stimuli are presented separately. Warmer colors indicate higher positive amplitudes. Electrode positions are marked as small circles; black dots indicate Pz.

We analyzed the EEG data using ERP analysis time‐locked to the onset of visual stimulus presentation. Across all three odor conditions, greater P300 amplitudes were observed with response to target stimuli than in response to the standard stimuli (Figure 2), particularly at the central–parietal sites within the 250–400 ms window (Figure 3).

P300 peak amplitudes within the 250–400 ms window were compared using a two‐way ANOVA (odor condition × stimulus type) at the Pz electrode (located at the midline parietal region of the scalp). Significant main effects were observed for odor condition, *F*(2, 36) = 3.30, *p* = 0.047, partial *η* ^2^ = 0.16, and stimulus type, *F*(1, 18) = 43.00, *p* < 0.001, partial *η* ^2^ = 0.71. Additionally, a significant interaction between odor condition and stimulus type was observed, *F*(2, 36) = 3.90, *p* = 0.029, partial *η* ^2^ = 0.18.

**FIGURE 4:**
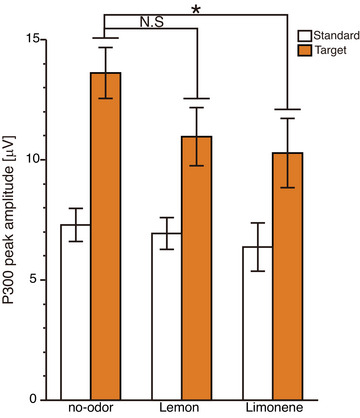
**Mean P300 peak amplitudes at Pz (located at the midline parietal region of the scalp) electrode**. Bar plots showing mean P300 peak amplitudes at the Pz electrode across odor conditions (no‐odor, limonene, and lemon essential oil) and stimulus types. Error bars indicate standard errors. Significant differences identified by post hoc tests are marked (*p < 0.05, N.S: not significant).

Post hoc Tukey's honestly significant difference tests were performed to assess pairwise differences between the no‐odor condition and each other odor condition. For the target stimulus, P300 peak amplitudes were significantly lower in the limonene condition than in the no‐odor condition (*p* = 0.018, 95% CI [0.40, 6.26], Cohen's *d* = 0.86; Figure 4). Conversely, the difference between the no‐odor and lemon essential oil conditions was not significant (*p* = 0.096, 95% CI [–0.28, 5.58], Cohen's *d* = 0.69). The differences in P300 peak amplitudes were analyzed by Bayesian analyses. The Bayes factors provided the evidence that P300 in the limonene condition was lower than that in the no‐odor condition (limonene < no‐odor: BF_10_ = 49.40). For lemon essential oil condition, the P300 reduction was moderate (lemon essential oil < no‐odor: BF_10_ = 6.37). Thus, Tukey's honestly significant difference and Bayes factor analyses both indicated that P300 in the limonene condition was reduced more than that in the no‐odor condition, although lemon essential oil's effect cannot be ruled out.

For the standard stimuli, no significant differences in P300 peak amplitudes were observed across odor conditions: no‐odor versus lemon, *p* = 0.93, 95% CI [–3.97, 1.89], Cohen's *d* = 0.25; and no‐odor versus limonene, *p* = 0.99, 95% CI [–4.13, 1.73], Cohen's *d* = 0.28.

Post hoc power analyses were performed to assess whether the sample size (*N* = 19) was sufficient to detect the observed interaction effects. The effect size for the interaction between odor and stimulus type on P300 amplitude was partial (*η* ^2^ = 0.18), corresponding to a medium‐to‐large effect according to Cohen's criteria (Cohen 1988). For the comparison between the limonene and no‐odor conditions under the target stimulus, the effect size was large (*d* = 0.86) (Cohen 1988), supporting the robustness of the observed effect despite the modest sample size.

### 3.2 Subjective Evaluations

Subjective odor intensity was assessed before and after each task block using a six‐point odor intensity scale of 0–5, where 0 = no odor, 1 = barely detectable (detection threshold), 2 = weak but recognizable (recognition threshold), 3 = easily noticeable, 4 = strong, and 5 = extremely strong (Table 1). Paired comparisons exhibited significant decreases in ratings after the first block for limonene and lemon oil as well as after the second block for lemon oil. However, average ratings across all conditions remained above level 2, corresponding to the recognition threshold. These findings indicate that despite partial habituation over time, participants continued to perceive the odor throughout the tasks. The intensity ratings were also assessed by two‐way ANOVA for within‐subject analysis (odor condition × assessment timing: before/after block 1 and 2). There was a significant main effect of the measurement timings (*F*(3, 54) = 17.98, *p* < 0.001). There was no significant main effect of odor condition (*F*(1, 18) = 0.71, *p* = 0.412) and no significant interaction (*F*(3, 54) = 1.53, *p* = 0.218).

Furthermore, after the second block, participants rated the congruency between the target color (orange) and the presented odor on a scale from –5 (completely different) to +5 (perfectly matching), along with their ratings of preference, pleasantness, and arousal. No significant differences were observed between limonene and lemon essential oil in any of these subjective measures (Table 2).

**TABLE 2:** Subjective ratings of odor properties.

Measure	Limonene (*M* ± SD)	Lemon (*M* ± SD)	Mean Diff	*t*	*p*	Cohen's *d*
Color congruency	2.89 ± 1.82	2.84 ± 1.86	0.05	0.09	0.930	0.03
Preference	2.83 ± 2.29	3.28 ± 1.23	−0.44	−0.62	0.550	0.27
Pleasantness	2.18 ± 2.27	2.35 ± 1.58	−0.17	−0.22	0.830	0.10
Arousal	1.33 ± 2.42	1.78 ± 2.49	−0.44	−0.49	0.630	0.19

*Note*: Mean ratings for color congruency, preference, pleasantness, and arousal across odor conditions. Color congruency was rated on a scale from –5 (completely different) to +5 (perfectly matching); other properties were rated on a six‐point scale from 0 (not at all) to 5 (extremely). Mean values were compared between limonene and lemon essential oil conditions by the paired *t*‐test.

## 4 Discussion

In this study, we examined whether citrus odors modulate visual attention, as indexed by the P300 component of ERPs and whether such modulation arises from the chemical properties of the odorants or from their semantic or emotional associations. To address this, we compared the effects of pure limonene and lemon essential oil during a visual oddball task and analyzed P300 amplitude responses.

Our results showed that P300 amplitudes evoked by target stimuli were significantly reduced under the limonene condition, particularly at the Pz electrode, while no significant reduction was observed in the lemon essential oil condition. Given that the P300 is closely associated with the attentional resource allocation, this finding indicates that limonene, as a single compound, exerts a chemically specific influence on selective attention. Conversely, lemon essential oil, despite its similar citrus‐like perceptual qualities, exhibited a non‐significant modulation compared to limonene.

Subjective ratings of odor–color congruency, preference, pleasantness, and arousal did not significantly differ between the limonene and lemon essential oil conditions. These findings support the interpretation that the modulation of P300 amplitudes observed following limonene exposure was attributable to the chemical properties of limonene rather than to hedonic valence or semantic associations. This aligns with our experimental objective of distinguishing chemically specific effects from those mediated by mood or perceptual congruency.

Notably, lemon essential oil contains both limonene and other compounds, such as α‐pinene, β‐pinene, γ‐terpinene, and myrcene (Cannon et al. 2015; Di Rauso Simeone et al. 2020; González‐Mas et al. 2019). Bayesian analysis did not rule out the inhibition effect from lemon essential oil, indicating that lemon essential oil exposure may also exert an influence on P300, although less reliably than limonene exposure alone. To match the perceived odor intensity, we applied a greater volume of limonene solution than of the lemon essential oil, resulting in a higher absolute concentration of limonene. Nevertheless, only the limonene condition led to a significant reduction in P300 amplitudes despite the presence of limonene in both odor types. This observation suggests that other constituents of lemon essential oil may counteract the attentional suppression effect of limonene, potentially through emotional modulation or interactive chemical effects. Further research is needed to isolate and examine the effects of the individual compounds within essential oils.

We observed reductions in P300 amplitudes following limonene exposure; however, the underlying mechanism warrants careful interpretation. The P300 amplitude is associated with selective attention (Polich 2007) and working memory load (Guerrero et al. 2022, Guerrero et al. 2023; Morgan et al. 2008; Ren et al. 2023). In this study, we interpreted the P300 component as primarily related to the allocation of selective attention, particularly the peak elicited in response to target stimuli in the oddball task. Therefore, the significant suppression of P300 amplitudes in the limonene condition likely indicates a reduction in selective attention to task‐relevant visual information.

One limitation of this study was the potential for olfactory habituation resulting from prolonged odor exposure through a nonwoven mask, which may have reduced the olfactory impact over time. We maintained a certain odor intensity; however, subjective ratings suggested a decrease in perceived odor strength before and after the experiment. Therefore, we cannot rule out the possibility that neural activity measured at the beginning and end of the experiment differed as a result of habituation. Implementing an intermittent odor delivery system that avoids continuous exposure could prevent habituation and enable a more accurate assessment of odor‐related brain activity.

## 5 Conclusion

Our findings offer new insights into how specific citrus odor compounds, including limonene, can modulate visual attention, as reflected by P300 changes. By comparing the effects of pure limonene and lemon essential oil, we demonstrated that attentional modulation is more likely attributable to chemical specificity rather than to semantic associations or affective responses. These findings enhance our contribution to a better understanding of olfactory–visual cross‐modal interactions and highlight the need to consider both chemical composition and task demands when assessing odor‐related cognitive modulation. Further research should investigate whether similar effects occur with other odor compounds, examine the neural mechanisms underlying these modulations, and explore their broader implications for cognitive performance in real‐world, multisensory environments.

## Author Contributions

**Kaori Tamura**: conceptualization, methodology, formal analysis, writing of the original draft, visualization, supervision, and funding acquisition. **Taiki Nishimura**: Investigation, data curation, formal analysis, and validation. **Yuko Ohno**: conceptualization, supervision, and writing – review and editing. **Shin'ichi Yoshimura**: conceptualization, supervision, and writing – review and editing. **Mitsuo Tonoike**: conceptualization, supervision, and writing – review and editing.

## Funding

This study was supported by a Grant‐in‐Aid for Scientific Research (KAKENHI) from the Japan Society for the Promotion of Science (Grant Number 23K11297). We used ChatGPT (Gpt‐4o, OpenAI) to assist with clear English expressions and sentences during manuscript preparation. The AI was not used to generate original content and analyze data or program. All scientific reasoning, analysis, and interpretation were conducted by the authors alone.

## Conflicts of Interest

The authors declare no conflict of interest

## Peer Review

The peer review history for this article is available at https://publons.com/publon/10.1002/brb3.71012

## Data Availability

The datasets collected and analyzed in the current study are not publicly available because of ethical restrictions and participant confidentiality, as approved by the institutional review board. The analytical code is available from the corresponding author upon reasonable request.
